# Winter is coming: the future of cryopreservation

**DOI:** 10.1186/s12915-021-00976-8

**Published:** 2021-03-24

**Authors:** Sanja Bojic, Alex Murray, Barry L. Bentley, Ralf Spindler, Piotr Pawlik, José L. Cordeiro, Roman Bauer, João Pedro de Magalhães

**Affiliations:** 1 School of Computing, Newcastle University, Newcastle upon Tyne, UK; 2 Biosciences Institute, Newcastle University, Newcastle upon Tyne, UK; 3 Department of Genetics, Faculty of Medical Sciences, University of Kragujevac, Kragujevac, Serbia; 4 Department of Chemistry, University of Warwick, Coventry, UK; 5 Faculty of Science, Technology, Engineering & Mathematics, The Open University, Milton Keynes, UK; 6 Magdalene College, University of Cambridge, Cambridge, UK; 7 European Biostasis Foundation, Riehen, Switzerland; 8 Cancer Genome Evolution Research Group, University College London Cancer Institute, University College London, London, UK; 9 World Academy of Art and Science, Napa, USA; 10 Department of Computer Science, University of Surrey, Guildford, UK; 11 Integrative Genomics of Ageing Group, Institute of Ageing and Chronic Disease, University of Liverpool, Liverpool, UK

**Keywords:** Cryobiology, Organ banking, Freezing, Vitrification, Biostasis

## Abstract

The preservative effects of low temperature on biological materials have been long recognised, and cryopreservation is now widely used in biomedicine, including in organ transplantation, regenerative medicine and drug discovery. The lack of organs for transplantation constitutes a major medical challenge, stemming largely from the inability to preserve donated organs until a suitable recipient is found. Here, we review the latest cryopreservation methods and applications. We describe the main challenges—scaling up to large volumes and complex tissues, preventing ice formation and mitigating cryoprotectant toxicity—discuss advantages and disadvantages of current methods and outline prospects for the future of the field.

## The origins of cryopreservation

Cryopreservation is the storage of biological material at low temperatures. Since ancient times, it has been known that biological material can be preserved longer at low temperatures. Indeed, archaeological findings indicate that as early as 2000 BC, icehouses were used throughout Mesopotamia to store foods [1]. The preservative effect of cold was also a topic of interest to the early experimentalists of the seventeenth century, most notably Boyle [2], who commented on the ability of ice to preserve human bodies and made several attempts to freeze and revive live animals, discovering species of frogs and fish that could survive encasement in ice.

Whereas Boyle could only speculate on the nature of cold, the intervening centuries have revealed its preservative effect lies in depriving biological systems of the thermal energy required for normal molecular motion and metabolism, in turn slowing cellular processes and decomposition. The ability to reliably generate the extremely low temperatures required for long-term preservation, typically below − 100 °C, came with the development of cryogenic technologies at the turn of the twentieth century. The modern cryopreservation of living systems, in the sense of biotechnology, can be traced to the discovery of the first effective cryoprotective agents (CPAs), otherwise known as “cryoprotectants”, in the 1940s [3]. Notably, Lovelock provided crucial early insights into the origins of cryoinjury and the action of CPAs [4, 5]. Later work by Mazur pioneered the application of quantitative models to describing cell changes during cooling [6] and paved the way towards theoretical approaches to studying cryopreservation.

The discipline of cryopreservation is now well established as a practical means of storing living cells and tissues and has grown to find applications throughout biology and medicine. As this review will highlight, cryopreservation now holds the potential to strongly benefit several areas of medicine by increasing the ease with which therapeutic cells, tissues, and organs can be stored.

## The challenge of low temperatures

Before discussing the medical applications and opportunities of cryopreservation, we should first consider the physiological effects of low temperatures, and the challenges they present.

Human cells have evolved to operate within an extremely narrow temperature range. Prolonged or extreme exposure to temperatures below the normothermic range of 36–38 °C is frequently harmful, as evidenced by hypothermia and frostbite. Despite this, sub-zero temperatures are not intrinsically pathogenic. The damage associated with low temperatures is, for the most part, the result of uncontrolled transitions between normothermic and low-temperature regimes. Therefore, the successful storage and revival of cells depend critically on the ability to induce and reverse low-temperature states in a controlled manner that minimises or ameliorates transition-related damage. Although this requirement is easy to state, it is in practice the central challenge of cryopreservation.

**Fig. 1:**
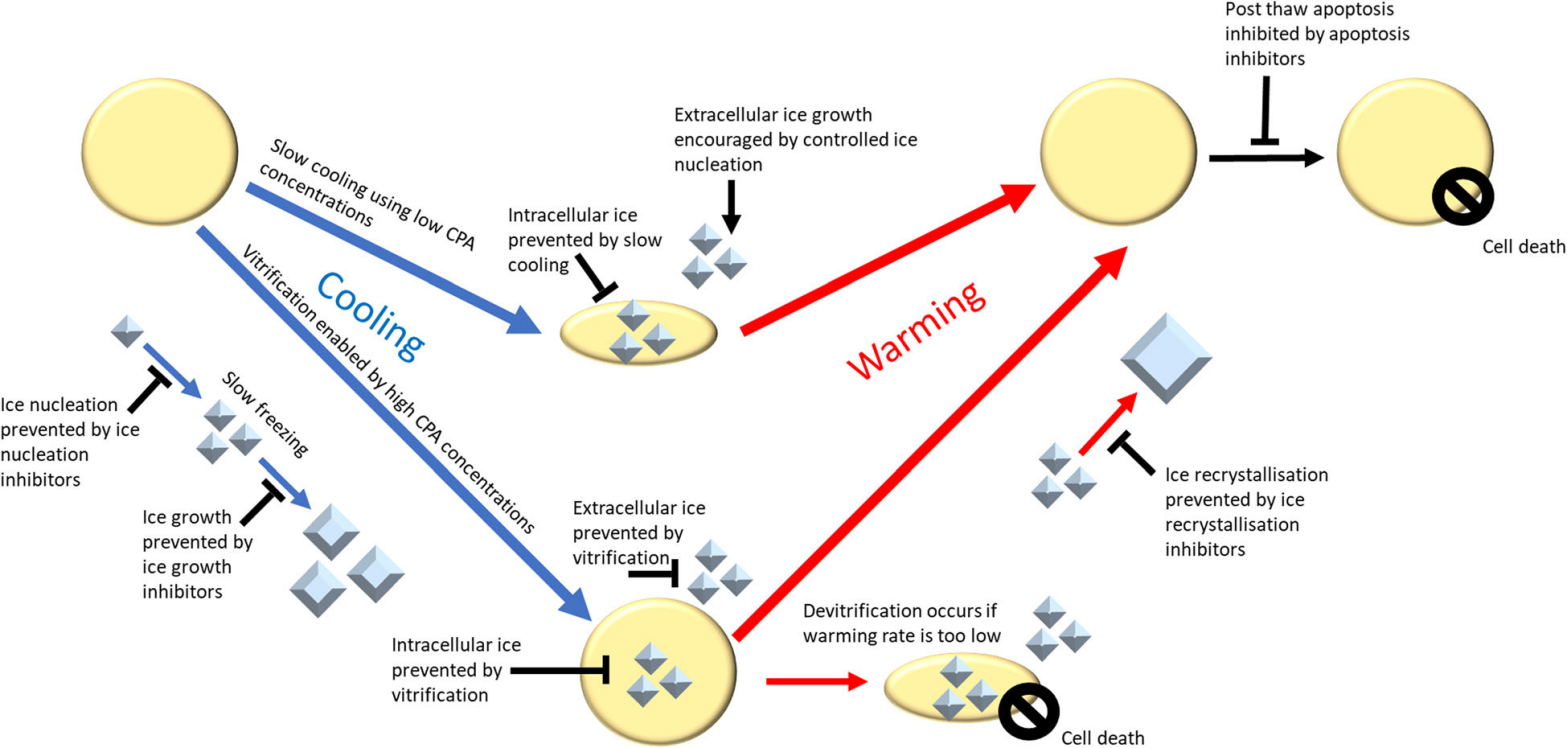
An overview of the mechanisms of cryopreservation and CPAs. Cryopreservation utilises either slow cooling, in which the sample is frozen at a controlled rate to allow water to flow out of the cell and prevent intracellular ice formation, or vitrification in which a high freezing rate and/or high CPA concentration prevents ice formation in the sample. Ice growth can be further controlled by ice nucleation inhibitors, controlled ice nucleation, ice growth inhibitors or ice recrystallization inhibitors. Devitrification occurs when a vitrified sample is warmed too slowly, resulting in ice formation. Apoptosis inhibitors may also be utilised to prevent cells from dying after cryopreservation from stress-induced apoptosis

How does the transition to low temperatures damage cells? Although numerous routes exist, much of the damage is driven, either directly or indirectly, by ice formation. In this context, *nucleation* denotes the first step in crystallisation as it initiates the growth of ice crystals. At typical rates of cooling, ice first forms in the extracellular spaces of tissues (Fig. 1). Ice excludes solutes, meaning that the extracellular solute concentration increases as water is removed from the extracellular fluid to form ice crystals. The resultant osmotic gradient across the cell membranes works to drive water out of the cells by osmosis, dehydrating cells and increasing the intracellular solute concentration. This increased solute concentration depresses the intracellular freezing point, preventing intracellular ice formation. This process can damage tissues through two routes: (1) solutes such as salts, which are maintained at safe concentrations under normal conditions, become more concentrated to induce osmotic stress [5]; (2) extracellular ice directly damages tissues, either by puncturing or crushing cell membranes or disrupting extracellular structures [7, 8]. However, the relative significance and interdependence of these two routes are still not entirely resolved.

If a sample is cooled too quickly for water to exit a cell, intracellular ice formation can occur. Intracellular ice crystals are much more damaging than their extracellular counterparts as they are capable of disrupting the cell’s internal structures, like lysosomes [9]. Intracellular ice formation is usually, but not always, lethal [10, 11]. Factors affecting lethality include the size of the ice crystals, their intracellular location, their mechanism of growth, their shape (cubic vs. hexagonal) and the warming rate [12–14].

To reduce the extent of ice-related damage, cryopreservation protocols usually employ CPAs [15]. CPAs act to reduce the aforementioned damage via a number of actions, many of which have not yet been fully identified. Some main effects are those of reducing the concentration of electrolytes [5] and also reducing ice formation by hydrogen bonding with water molecules to prevent them from bonding to ice crystals [16]. CPAs capable of crossing cell membranes are of particular value. The use of such molecules, termed penetrating CPAs (pCPAs), can substantially reduce cryoinjury. The discovery of pCPAs dates back more than 70 years to when the cryoprotective effects of glycerol were discovered. In their landmark study, Polge et al. discovered that glycerol improved the revival of spermatozoa from sub-zero temperatures [17]. Glycerol remains a commonly used pCPA, with other notable examples being propylene glycol (PG), ethylene glycol (EG) and dimethyl sulfoxide (DMSO).

The cellular protection afforded by CPAs has made the cryopreservation of cells and simple tissues a routine procedure; however, there are still several challenges to be surmounted in the preservation of complex tissues and organs. Work to categorise these challenges has identified six broad areas of need [18]. These include: (1) improved control over ice formation; (2) methods to reduce CPA toxicity, to allow their use at higher concentrations; (3) the elimination of thermo-mechanical stresses; (4) methods to prevent or reverse ischemic injury prior to or following preservation; (5) the prevention of chilling injuries; and (6) the development of safe protocols for organ revival. These will be discussed in the following sections, along with the initial progress towards their solutions.

Here, we first review the importance of medical cryopreservation followed by the major cryopreservation methods, including slow cooling and vitrification and discussing their strengths and weaknesses; then, we discuss the key specific aspects of cryopreservation and tissue damage. Lastly, we discuss the challenges and avenues for future research.

## The medical need for cryopreservation

### Organ and tissue preservation

Cryopreservation has multiple and important applications, particularly in medicine. The fact that it can significantly slow down all biochemical reaction kinetics renders cryopreservation highly attractive as a means to preserve organs and therefore facilitate the transplantation process. The lack of organ availability constitutes a major challenge and a significant medical burden for society. According to the World Health Organization (WHO), only 10% of the worldwide need for organ transplantation was met in 2010 [19]. The lack of transplantable organs stems partially from a shortage of suitable donated organs, but more importantly from the lack of preservation capability. Although the number of transplanted organs is much lower than what is actually needed worldwide, it was estimated that approximately two thirds of potential donor hearts are discarded [20]. Moreover, up to 20% of potential donor kidneys end up being discarded in the USA [21, 22], and up to 50% of donated pancreases in the UK [23], primarily due to the limited time available to find suitable recipients. This extensive waste could be largely prevented by increasing the time through advancing preservation methods [24], especially cryopreservation, which is currently the only true long-term preservation solution. The potential of organ and tissue preservation to transform medicine has recently been reviewed by Giwa and co-workers [25].

The growing shortage of donor organs has become a central problem limiting the effectiveness of transplantation. Advancing preservation technologies can ultimately benefit many thousands of people worldwide by increasing local and global access to transplantation, by improving transplantation outcomes and stimulating progress in related areas such as immune tolerance induction and xenotransplantation [26]. Prolongation of preservation times would enable transportation of organs to wider geographical areas, comprehensive evaluation of donor tissues and better graft characterisation [27]. Notably, the potential public health impact of improved organ banking capability would benefit many areas of medicine by the banking of organs as well as other complex tissues, improving not only transplantation but also tissue engineering, trauma medicine, oncofertility, basic biomedical research and drug discovery [18].

### Pharmaceutical research

The ability to bank large quantities of tissues and cells is of immense interest for the discovery, development and evaluation of drugs. In particular, drug discovery and testing could benefit from cryopreservation of tissue slices. Tissue slices represent a powerful in vitro tool for studying biological processes as they closely resemble the organ from which they were derived, containing all specific cell types present in the organ. Much effort has been put into optimising the preparation and culturing techniques of various tissue slices such as liver, brain, intestinal and kidney slices [28–31]. Under optimal conditions, tissue slices can be kept viable for at least 24 h after thawing [32]. The cryopreservation of tissue slices greatly facilitates their use in pharmaco-toxicological research, leading to efficient use of human organ material and a decrease of laboratory animal use [32].

Human tissues grown from stem cells are also of high interest for the pharmaceutical industry. They can enable large-scale drug screening tests on human tissues, facilitate the identification of adverse effects of drugs and thus revolutionise toxicology screening [33] and drug discovery [34, 35]. Given the costs associated with drug development [36], improved cryopreservation capability would also constitute an advance for the pharmaceutical industry. For animal welfare, the scenario of using lab-grown tissues additionally constitutes an attractive alternative.

## Cryopreservation methods

### Freezing vs. vitrification

**Fig. 2:**
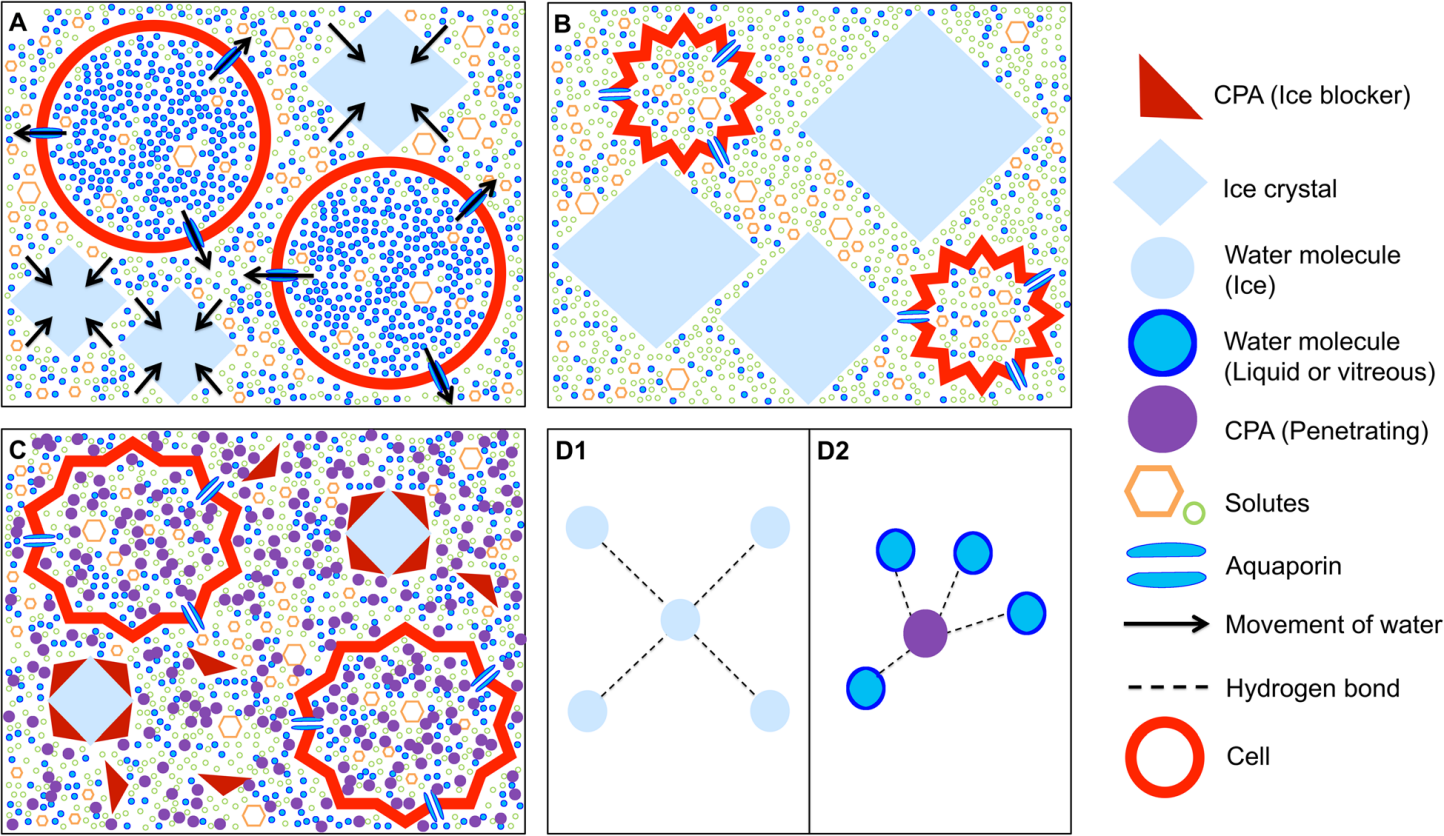
How cryoprotective agents (CPAs) work. **A** When a sample is cooled, ice first forms in the extracellular space. Ice excludes solutes, so the extracellular solute concentration increases as extracellular water becomes part of the ice crystals. Intracellular water is then drawn out of the cells via osmosis. **B** In unprotected cells, the intracellular solute concentration increases and causes damage. **C** Penetrating CPAs permeate the cell membrane and increase the intracellular solute concentration which prevents water loss and dilutes other solutes inside the cell which can cause damage at high concentrations. Ice blockers bind to ice crystals, preventing them from growing, or to nucleators, which prevents heterogenous ice nucleation. **D1**, **D2** Penetrating CPAs interfere with homogenous ice nucleation by colligative interference, which depresses the freezing point of the solution. **D1** Water forms a regular ice lattice. **D2** A CPA molecule disrupts the hydrogen bonding between water molecules

Cryopreservation has become a well-established method for preserving cells and tissues [37], including sperm, oocytes and ovarian and embryonic tissue which are now cryopreserved on a large scale. Currently, the main approaches for cryopreservation are freezing (i.e. the liquid phase changes to a solid crystalline phase) and vitrification (i.e. the solidification to a glass-like state without ice formation), as illustrated in Fig. 1. The methods of freezing and vitrification are commonly distinguished by whether during sample-cooling ice crystal growth occurs or is suppressed, respectively (Fig. 2). Notably, freezing is applied for a wide range of sample volumes, whilst vitrification usually has a small application size.

**Fig. 3:**
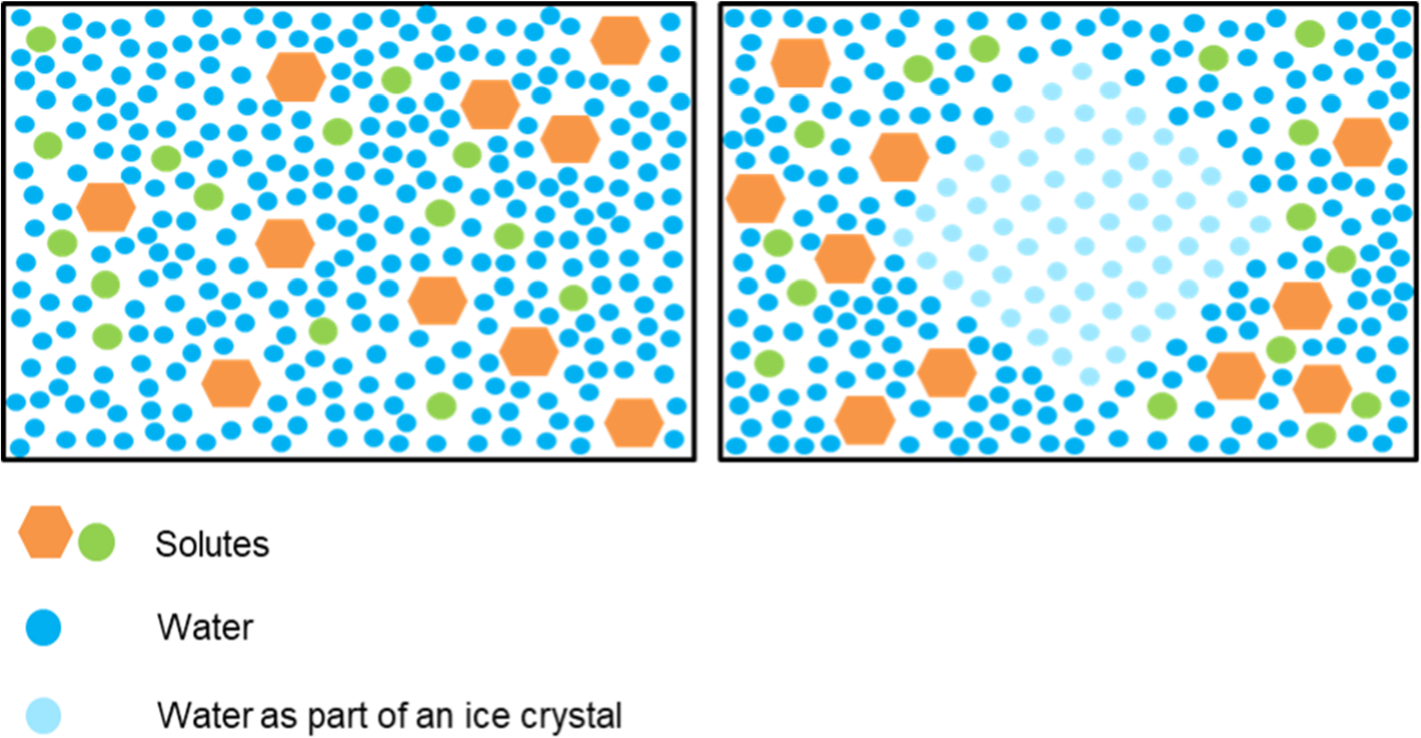
A vitrified substance (left) is formed when liquid transitions into a highly viscous, glass-like state that prevents translational molecular motion. In vitrification, molecules remain in the position they were in when the substance was vitrified. This is different to freezing (right), in which ice crystals progressively grow as the temperature is decreased, excluding solutes and thus causing the solute concentration to increase

In freezing, a too low cooling rate can give rise to cryoinjury due to solution effects. On the other hand, the rate needs to be slow enough so that there is sufficient time for the water to exit cells, to prevent intracellular ice formation. Freezing usually involves slow cooling protocols with cooling rates of approximately 1 °C/min [38]. However, different cell types have different optimal cooling rates [39].

Vitrification is a separate physical process from freezing and occurs at the so-called “glass transition temperature”, around − 80 to − 130 °C. In vitrification, samples solidify with no ice crystal formation (Fig. 3). It has been previously shown that the vitrification of small biological samples can be achieved without CPAs using extremely high cooling rates, e.g. with human sperm [40]. In larger living systems where an extremely high cooling rate cannot be achieved, a large percentage of the water in the sample must be replaced by a vitrifying CPA to avoid the damaging effects of ice formation. Vitrification ultimately forms a stable glass-like state, preserving the molecular contents indefinitely [41].

Importantly, warming after cryopreservation can lead to injury due to ice crystal formation, which can occur both after freezing or vitrification. Along these lines, “ice recrystallization” denotes this damaging process of large ice crystal growth during thawing of frozen cells or tissue. Notably, and somewhat counterintuitively, “devitrification” denotes the process of crystallisation during warming and after vitrification. Hence, devitrification does not mean the process of warming of a vitrified solution, but the formation or recrystallization of ice crystals during rewarming.

In vitrification, CPAs are primarily used to inhibit ice formation by depressing the freezing point of the solution and increasing its viscosity and the glass transition temperature. In this way, CPAs decrease the critical cooling rate for vitrification (*V*_ccr_) [42], allowing the sample to solidify before ice formation can occur [43]. Notably, CPAs can also impact on the critical warming rates and so reduce the risk of devitrification. What are the requirements for tissues to vitrify and undergo a transition into the glass-type state? Most importantly, the cooling rate has to be sufficiently high, so that ice formation is prevented. Notably, vitrification requires very high concentrations of CPAs to lower *V*_ccr_ to practical levels. Unfortunately, the toxic impact on cells caused by these concentrated CPAs is problematic [44]. This problem can to some extent be mitigated, as discussed ahead in the "Cryoprotective agents (CPAs)" section.

Although slow cooling and vitrification are well-established and are used for various applications, they each have their advantages and disadvantages. For instance, the recording of slow cooling protocols is practical which facilitates the demonstration of compliance with standard operating procedures and Good Manufacturing Practice. However, it often requires relatively costly equipment [45]. Moreover, although slow cooling is more time consuming, this apparent disadvantage can entail a more homogeneous distribution of CPAs [46], which need to diffuse through and permeate the extracellular space to take full effect. Also, the slower and more controlled cooling process in slow cooling is better suited for quantifiable and reproducible protocols. The temperature dynamics within the sample volume are therefore more amenable to modelling in a quantitative manner. However, extracellular ice formed during slow cooling can also have adverse effects. It remains to be investigated how such damage depends on cooling and thawing rates, as well as CPAs.

Rapid cooling to below the glass transition temperature and/or rapid warming from below the glass transition temperature during the warming process may induce fracturing of the glass in which the biological system is embedded [43, 47, 48]. Cell-type or tissue-specific cooling and warming rates can potentially avoid the problems of devitrification and recrystallisation, and further research is required to elaborate on such solutions (more information can be found in Fahy and Wowk, 2015, p. 45). Notably, such highly adapted protocols can be problematic with regard to the cryopreservation of organs comprising different cell or tissue types.

Table 1 summarises the key differences between these main approaches. Importantly, there is no gold standard in cryopreservation, and different labs often employ their own cryogenic processing protocols that might substantially differ from other labs’ approaches.

**Table 1:** Comparison of typical key aspects of slow cooling and vitrification

Feature	Slow cooling	Vitrification
Duration	Long (h)	Short (s)
Usage of CPAs	Low concentrations	High concentrations
Costly components	Equipment	Operation
Controllability/reproducibility/recordability	High	Low
Operation skills required	Low	High
Main risks	Extracellular ice formation, osmotic damage	Intracellular ice formation, CPA toxicity, fracturing, osmotic stress

### Directional freezing and directional vitrification

**Fig. 4:**
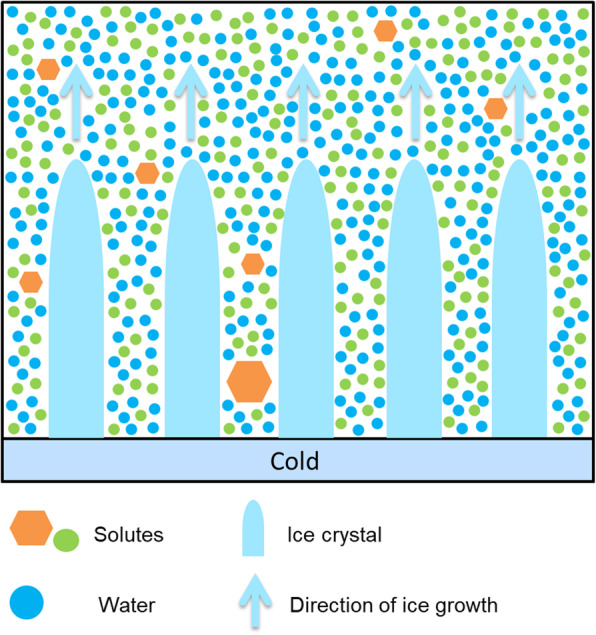
In directional freezing, cold is applied to one side of the sample causing a temperature gradient. Uniform ice lamellae form along this gradient with heat being conducted through the unfrozen part of the sample

Directional freezing (also called directional solidification) is a method in which a temperature gradient is used to cause the progressive growth of ice along the temperature gradient (Fig. 4). This can be achieved by moving the sample along the gradient at a controlled velocity or by placing the sample between two thermo-conductive blocks. In conventional freezing, the latent heat of fusion released during freezing is conducted through the frozen portion of the sample, this may cause it to melt and can result in repeated freeze-thaw cycles which can cause increased cellular damage. The morphology and rate of ice growth are uncontrolled and non-uniform. In directional freezing, heat is removed by conduction through the unfrozen portion of the sample, preventing damage by freeze-thaw cycles. The directional ice growth results in the formation of lamellae with cells trapped between them, reducing mechanical damage [49]. Directional freezing also allows for faster cooling without inducing intracellular ice formation [50].

Directional freezing has been used to cool a rat’s hind limb to − 140 °C before submersion in liquid nitrogen (LN_2_). The hind limbs were then successfully revived and re-transplanted onto the rats. Whilst these limbs were non-functional due to the motor neurons having been severed during amputation, the limbs were otherwise viable [51]. Directional freezing has also been used for the successful cryopreservation of a pig’s liver at − 20 °C. The liver was later revived and attached to another pig by auxiliary transplantation and demonstrated normal bile production and blood flow [52].

A related technique is directional vitrification; this is achieved using the same principle as directional freezing, but with a very high cooling rate. Information on directional vitrification is limited; however, this technique may allow for vitrification with cryoprotectant concentrations as low as 17.5% PG [53].

Both directional freezing and directional vitrification are rich avenues for future research. Further work on directional freezing should aim to further lower the temperature to which organs can be frozen to − 130 °C, the temperature at which storage without degradation can be achieved for centuries or millennia [54].

## Key aspects of cryogenic processing protocols

### Cooling

As mentioned above, during slow cooling, extracellular water freezes first and cells lose water due to osmotic pressure, with more solute outside than inside the cells. During fast cooling, the transmembrane transport of water is not fast enough, and the intracellular water is more likely to freeze as well [55]. It is a complex problem to determine how quickly to best cool cells and tissues: the optimal cooling rate minimises the dangers of injury due to high solute concentrations as well as intracellular ice formation. This “sweet spot” depends on various aspects, including biophysical cell membrane properties, the volume of the sample, the CPA type and the concentration.

When cooling a macroscopic sample, e.g. a 2-mL cell suspension, the large heat capacity of water causes the core of the sample to cool more slowly than the surface. The sample therefore usually nucleates in the surface region first because it is closer to the colder cryovial wall. The release of the latent heat will then further increase the temperature lag between rim and core temperatures. For vitrification protocols, the cooling rate needs to be slow enough to achieve a homogeneous temperature distribution, especially around and below the glass transition temperature to not cause cracking [56].

### Warming

It has been established that the quality of a biological sample after cryopreservation depends not only on the method of cooling, but also on the warming of the sample. (Note, we use the word “thawing” only in the context of freezing. In vitrification, no ice crystals are formed, and so the cells or tissues are technically not frozen. Hence, it is more appropriate to use the word “warming”). During warming, ice crystals can grow around an initial nucleus. Both ice nucleation and growth depend on temperature, yet in a different way. The process of ice *growth* is fastest just below the freezing point and attenuates when the temperature drops. The nucleation reaches the peak well below the freezing point. In other words, the cooled sample first passes through an ice growth zone (but with few nuclei available to seed the growth), then, when the temperature for crystallisation is no longer optimal, enters the zone of nucleation. For this reason, rewarming the sample brings more risk of recrystallisation and devitrification—when the sample enters temperatures optimal for freezing, it is already well nucleated [43]. This is also the reason why the critical rates of cooling and rewarming necessary to outpace the ice formation are different: the former must deal with few nuclei, the latter with many [43]. Notably, multiple small crystals (100–300 nm) [57, 58] are much better tolerated than a few large ones. In this context, it was demonstrated that the addition of a temperature hold during rewarming at the glass transition temperature can significantly alter the impact of physical forces during warming for certain cell types [59]. It remains to be investigated how to optimally choose the dynamics of temperature changes during cooling and warming, in a tissue-specific manner.

Recently, a novel method for warming has been tested with promising results. Manuchehrabadi et al. demonstrated that inductive heating of magnetic nanoparticles can be employed to improve tissue cryopreservation [60]. Other methods [61–65], like electromagnetic heating [60, 66–68], have also been proposed.

### Nucleation temperature

The initial formation of ice crystals is only possible after a primary nucleation event has occurred. If uncontrolled, such primary nucleation is stochastic [69]. This means, for example, when a specific aqueous sample is cooled at a given cooling rate, it is not deterministic at what time or temperature it will start freezing. The temperature at which primary nucleation occurs within a sample is called the nucleation temperature [70]. It has been shown that the nucleation temperature affects cell survival in a similar way as the cooling rate does, namely, a too high nucleation temperature can be detrimental to cells caused by solution effects, whilst a too low nucleation temperature, close to the spontaneous nucleation temperature, can cause intracellular ice formation [70]. The highest survival of frozen cells was found at intermediate nucleation temperature regimes [71]. It was further found that at optimal intermediate nucleation temperatures the concentration of CPAs could be reduced. In order to reduce the effects of toxic CPAs, the active control of the nucleation temperature has been proven to be beneficial for cell survival after thawing.

There are various methods for actively controlling the process of nucleation for slow cooling [72, 73]. Briefly, the nucleation temperature can be controlled by creating locally supercooled conditions, such as via the application of cooled metal rods, expanded gases [74] or Peltier elements [75, 76]. Furthermore, ice-nucleating agents (INAs) exist that can directly induce nucleation events, with examples including silver iodide, ammonium sulfate, long-chain alcohols and nanoparticles [77]; however, as with CPAs, the utility of these is often limited by their toxicity, availability or biocompatibility. Promisingly, high-activity INA proteins have been discovered in multiple frost-tolerant organisms, with the best characterised being those from epiphytic bacteria of the genera *Pseudomonas* and *Erwinia* [78–81]. The probability of a primary nucleation can be increased by electrofreezing, where a high electrical field is locally applied to a sample [70, 76]. Other physical methods to control nucleation include the use of ultrasound [82] or laser light pulses [83], reviewed elsewhere [73].

### Cryoprotective agents (CPAs)

As mentioned above, CPAs can prevent cryoinjury in multiple ways, including the reduction of solution effects [5, 55]. A good CPA should possess the following properties: it should be water-soluble at low temperatures and to high concentrations, have good penetrance of cell membranes and have low toxicity. The concentration-dependent mechanisms of how pCPAs like DMSO and EG benefit cryopreservation has been investigated through simulations and experiments, which have suggested that at intermediate CPA concentrations, pore formation occurs within the membranes which increases the hydraulic membrane permeability [84, 85].

Some macromolecular CPAs like trehalose, dextran, sucrose or hydroxyethyl starch, which do not permeate cell membranes, are suggested to stabilise proteins by the “preferential exclusion” effect [86]. It was found that they prevent freezing damage best in combination with pCPAs like DMSO [87]. Slow cooling studies revealed that trehalose can alter the ice/cell interaction which can improve post-thaw cell survival [88].

The usage of DMSO as a CPA was first described by Lovelock and Bishop [4], 10 years after Polge et al.’s work on the cryoprotective properties of glycerol [17]. DMSO offers the advantage of enhanced permeability vs. glycerol for many cell types [89]. Currently, DMSO is the most commonly used CPA for the storage of stem cells [90]. However, the infusion of stem cells cryopreserved with DMSO has been associated with toxic reactions such as vomiting, cardiac dysfunction, arrhythmia and others [90, 91].

Despite these advances in the identification and use of CPAs, CPA toxicity remains the main challenge in cryopreservation [16]. Whilst the mechanisms of CPA toxicity are only partially understood, some mechanisms have been elucidated and classified as either *specific* or *non-specific* toxicity. *Specific toxicity* refers to CPA damage caused through a mechanism unique to that particular CPA. For example, EG is metabolised in the liver to glycolic acid, causing metabolic acidosis [92]. *Non-specific toxicity* refers to damage that occurs due to properties that CPAs have in common. For example, CPAs form hydrogen bonds with proteins; however, their bonding strength is higher than that of water, with the potential to denature proteins at high concentrations [93].

Importantly, the toxic effect of CPAs increases with temperature. Loading ramp protocols where CPAs are administered at increasing concentrations as the sample is cooled can greatly improve post-warm viability. Another reason for using a loading ramp is to slowly equilibrate the sample with the CPA to reduce osmotic stress [43]. Indeed, the potentially lethal mechanical stresses on cells due to CPA administration and removal are a major current concern in cryobiology, particularly for vitrification [94]. Moreover, nonequilibrium concentrations of CPAs between regions of the sample [95] or across the membrane [96, 97] can reduce the success of cryopreservation.

#### Biological antifreeze proteins, compatible solutes and ice blockers as alternatives

Some animals are known to produce substances that allow them to survive cold temperatures by avoiding ice formation [41, 98–100]. These substances can be either cryoprotectants (e.g. glycerol) or antifreeze proteins. The latter can bind to ice and hinder its growth [43]; however, the use of such proteins is limited by their high costs. Synthetic alternatives are used in research. These include polyvinyl alcohol (PVA) as an ice growth inhibitor, which binds to and prevents ice crystals from getting larger, and polyglycerol (PGL) as an ice nucleation inhibitor, which prevents ice from nucleating in the first place (see Fig. 1) [43, 101, 102]. They are effective even in small quantities [101] and widely used (examples [29, 103–108].

Compatible solutes are small organic osmolytes like hydroxyectoine, ectoine and L-proline which are produced by microorganisms under stress. These have been used to reduce the amount of pCPAs in freezing protocols [109, 110]. There may be potential benefits in using compatible solutes for vitrification methods.

#### Vitrification solutions

Vitrification solutions are used to decrease *V*_ccr_, allowing larger samples to be vitrified. Pure water can be vitrified if it is cooled fast enough, but to cause this, extremely high cooling rates (in the order of 10^6^ °C/s) are needed. To counter this problem, high CPA concentrations can be used to lower *V*_ccr_ to a level achievable in practice by plunging a sample into LN_2_. However, samples with a high volume to surface area ratio exhibit a lower internal cooling rate [111, 112].

To preserve larger tissues with a higher volume to surface area ratio, vitrification solutions need to be made more concentrated, or otherwise adapted to further lower the *V*_ccr_. This often comes at the cost of increased toxicity. As such, the development of vitrification solutions is dependent on finding new CPAs or combinations of CPAs that decrease *V*_ccr_ whilst having lower levels of toxicity [43]. A number of advances in vitrification solutions have been made in recent years, as follows.

##### Neutralising toxicity

The simplest vitrification solutions are high concentrations of single CPAs such as EG or DMSO, which are toxic at high concentrations. It has been found, however, that different CPAs can be combined in a single solution to reduce toxicity. By using a solution of different CPAs, it is possible to avoid using any one CPA at a concentration that would cause specific toxicity. Additionally, certain CPAs can directly neutralise the toxicity of other CPAs, for example, DMSO neutralises the toxicity of formamide, allowing for a higher total concentration of CPAs to be used with reduced toxicity. This allows for a higher total CPA concentration for the same level of toxicity [44, 104].

##### Ethylene glycol

It was found that using ethylene glycol in vitrification solutions in place of PG reduces the non-specific toxicity of the solution. This is because EG forms weaker hydrogen bonds than propylene glycol, meaning that macromolecules are protected by increased hydration from the greater number of remaining water molecules [16, 104].

##### Non-penetrating CPAs (npCPAs)

As discussed in "The challenge of low temperatures" section, ice can form more readily in the extracellular space than in the intracellular space because the intracellular space has a higher concentration of solutes [16]. For this reason, a lower concentration of cryoprotectants is needed inside the cell. High-molecular weight polymers that do not penetrate cell membranes, such as polyvinylpyrrolidone (PVP), can be used to mimic the effects of intracellular solutes in the extracellular space, meaning that a lower concentration of the more toxic penetrating cryoprotective agents (pCPAs) is needed for vitrification [7]. npCPAs also reduce chilling injury as the resulting solution is hypotonic. 

##### Synthetic ice blockers

Ice blockers are another kind of npCPA which bind directly to ice and to heterogeneous ice nucleators. These are the synthetic equivalent to anti-freeze proteins [7]. Non-penetrating ice blockers such as polyvinyl alcohol (PVA) are a cheaper and more effective alternative to animal-derived antifreeze proteins. Adding ice blockers in small amounts inhibits heterogeneous ice nucleation [43, 101].

##### Methoxylation

M22 is an advanced proprietary vitrification solution created by the company twenty-first Century Medicine (21CM). The solution, described in Fahy et al. [113], contains the methoxylated polyol 3-methoxy-1,2-propanediol, which has increased cellular permeability and decreased viscosity. This decreased viscosity is important for vitrifying larger tissues and organs because it increases the ability of the CPA to perfuse into tissues. Additionally, 3-methoxy-1,2-propanediol increases the glass transition temperature of tissues and decreases the critical warming and cooling rates of the solution [114, 115].

##### Hypertonicity

Chilling injury can be reduced by making hypertonic vitrification solutions, which have an increased concentration of non-penetrating components [113]. Whilst the reasons for the protective effect of hypertonicity are poorly understood, it has been suggested that it reduces damage due to the thermal contraction of cell membranes [116].

## Current applications of cryopreservation

### Organ preservation and banking

As mentioned in the “Organ and tissue preservation” section, many thousands of lives worldwide would benefit if replacing organs and tissues on demand became a reality [25]. Some of the approaches to extend organ lifetime are ex vivo continuous perfusion at normothermic temperatures (35–37 °C) and a combination of perfusion with hypothermic temperatures (4–10 °C), which is routinely used for kidney transplants and can extend lung survival times from 8 to 21 h [117]. The addition of CPAs and suppression of ice formation (supercooling) in rat livers enabled perfusion on even lower temperatures (− 6 °C), extending storage times to 4 days [118]. However, so far, there are no effective methods to reliably preserve solid organs beyond 3–12 h [119–121]. Not only days or weeks but even hours of additional preservation time would greatly increase the number of possible transplantations.

Kidneys and hearts have been the most widely studied organs, but neither has been consistently recovered after cooling to temperatures lower than − 45 °C. Nevertheless, sporadic survival of kidneys has been claimed after cooling to lower temperatures [95, 113]. Along those lines, Fahy et al. reported success in vitrifying a rabbit kidney at − 130 °C which was rewarmed using a special conductive warming technique combined with perfusion. After warming, the kidney was transplanted into a recipient rabbit that lived for 48 days with a working kidney before being sacrificed [95]. More recently, Marco-Jimenez et al. succeed in creating a long-term biobank of kidney precursors as a potentially unlimited source of organs for transplantation [122] as well as cryopreserving renal primordia able to developed morphologically normal glomeruli after warming and allotransplantation [123].

### Tissue and cell banking

#### Corneal tissue

According to the WHO, 10 million people worldwide require surgery to prevent corneal blindness as a result of trachoma, with a further 4.9 million suffering from total blindness due to corneal scarring [124]. There is a considerable discrepancy between the supply and demand of transplantable corneas [125]. Even though currently the main problem in most countries is the insufficient availability of transplantable corneas, corneal cryopreservation would contribute to an amelioration of the situation. Moreover, corneal tissue engineering is a highly researched topic and has the potential to increase the supply. Corneal cryopreservation would offer an unlimited storage time and eliminate the time-dependent deterioration [126]. Human corneas for transplant are commonly stored at 2–8 °C for up to 14 days, or by organ culture, which allows up to 4 weeks of storage [127]. The first cryopreservation technique developed for rabbit, cat and human corneas used 7.5% DMSO and 10% sucrose. Although cryopreserved corneas have been successfully transplanted, significant endothelial damage occurred as a result of freezing [128]. Cells in monolayers, such as corneal endothelium, are more susceptible to freezing injury compared to isolated cells frozen in suspension. In human corneas, endothelial cells have a very limited proliferative capacity, and cells lost during cryopreservation are not subsequently replaced by mitotic division. Well-preserved corneal endothelium is therefore crucial to the successful outcome of full-thickness corneal transplantation. A number of attempts have been made to improve the reliability of corneal cryopreservation. Rabbit and human corneas have been vitrified using 6.8 M propane-1,2-diol and shown to retain endothelial function [128]. Although this technique showed some success, it requires exposure to very high concentrations of solutes and is very time-consuming. Recently, a cryopreservation protocol involving interrupted slow cooling at 1 °C/min to a plunge sub-zero temperature (− 35 °C for porcine and − 45 °C for human) before storage in LN_2_, and using a combination of a pCPA (5% DMSO) and a npCPA (6% HES), has been successfully applied to cryopreserve corneal endothelial cells [126]. So far, however, cryopreserved human corneas have not been adopted by eye banks for routine use due to their significantly lower performance compared with fresh corneas.

#### Retinal pigmented epithelium (RPE)

Transplantation of RPE may have a potential clinical application for the treatment of RPE-specific retinal degeneration such as age-related macular degeneration (AMD). AMD is the most common cause of blindness in the developed world, affecting one in three people by 75 years of age [129] with an estimated prevalence of ∼600,000 significantly visually impaired people in the UK and over 8 million worldwide [130, 131]. The development of a suitable storage method for RPE is necessary for the establishment of future RPE replacement therapy [132]. The feasibility of an RPE storage bank has been investigated by Durlu and Tamai who performed transplantation of viable cryopreserved RPE cells into the subretinal spaces of adult albino rabbits and 23-day-old rats [133]. Successful transplantation was confirmed postoperatively by light and electron microscopy. In rabbits, xenografted RPE cells residing on Bruch’s membrane of the host retina were disclosed without any morphologic difference between the fresh and cryopreserved RPE cells in situ at day 25 following transplantation [133]. In rats, subretinal injection of cryopreserved RPE cells partially rescued photoreceptor cells locally at the transplanted area as observed 3 months postoperatively [133]. Together, their data confirmed that cryopreserved RPE cells can be used for RPE transplantation.

Although changes in viability, altered cellular functions and impaired biological activities caused by cryopreservation are usually transient, and cells resume their activities with time or after repeated passage, some irreversible changes have also been reported. For example, Basu et al. found an increase in abnormal chromosomes in cultured bovine RPE cells after freezing, which further increased after cell passage [134]. Another study investigating RPE cell lines suggested that inappropriate freezing conditions may not only decrease cell viability but also induce replicative senescence [135]. Valtink et al. also demonstrated that RPE cells can be cryogenically stored and kept available for transplantation for a prolonged period of time [136].

#### Retinal Müller glial cells cryopreservation

Müller cells are the dominant macroglia of the vertebrate retina [137]. In addition to their various important functional roles in retinal physiology, they are also involved in retinal pathology, for example, the cells display altered membrane properties in cases of proliferative vitreoretinopathy [137, 138]. The use of human tissue is limited by reasons of availability, thus developing a method for long-term storage of these cells is desirable. Biedermann et al. described a cryopreservation method in which dissociated Müller cells are stored in 10% DMSO. They showed that the main electrophysiological properties were not altered by this method and that voltage- and ligand-gated currents can be recorded from cryopreserved cells even after 2 years of storage [139].

#### Nerves and brain tissue

The replacement of damaged or missing cells is an emerging treatment strategy for a number of neurological disorders. The cryopreservation of neural cells and tissues has not been applied with sufficient success to enable it to be incorporated into routine clinical practice. Isolated brain tissue has a short shelf-life and cells are usually used for experiments immediately after isolation or refrigerated for a short period of time before use. Storage at 4 °C allows good survival of neural tissue up to a maximum of 8 days [140–142]; therefore, developing long-term storage procedures is of vital importance. Cryopreservation of primary neural cells would also greatly facilitate basic neuroscience research [143]. The ability to cryopreserve brain tissue would also facilitate pooling of tissue from multiple donors, allowing plenty of time for necessary testing to ensure safety and function of the cells prior to transplantation. Successful cryopreservation would be beneficial for neural tissue engineering too, allowing long-term storage for purposes of inventory control, quality control and product distribution [144].

The first attempts to achieve nerve tissue cryopreservation were published in 1953, by Luyet and Gonzales, who demonstrated that chicken brain tissue survived freezing to − 196 °C, following exposure to EG and rapid cooling [145]. Since then, different studies have suggested methodologies to cryopreserve neurons and neural stem cells [146–154] as summarised by Paynter [147].

Many studies have measured the qualitative outcome of viability [149, 150, 154] or cell membrane integrity [152]; however, only a few authors have characterised cryopreserved cultures in deeper detail. Post-thaw viability (based on membrane integrity assays) does not necessarily reflect the quality of the cryopreservation procedure and proper functionality tests must be carried out. For example, Higgins et al. provided a qualitative evaluation of neuritic tree formation [151] whilst Quasthoff et al. included a parametric analysis of morphological and functional development of the cultures [153].

In addition, cryopreservation of organised adult cerebral tissue slices is of potential interest for pharmaceutical neuropsychiatric drug evaluation and development. Pichugin et al. showed that the viability and the structure of mature organised, and complex neural networks can be well preserved by vitrification [29].

Cryopreservation of single neural cell suspension has also been described, like neural progenitor cells [151, 154–157] and neurally differentiated pluripotent stem cells [158]. Moreover, cryopreservation of brain spheroid cultures is another emerging topic [148, 159, 160]. For example, Ma et al. compared the survival rate and viability after cryopreservation between three different states of neural stem cells (NSC): single-cell suspension, NSC spheres with diameters of 30–50 μm and 80–100 μm. NSC spheres with a diameter of 80–100 μm achieved the best survival rate of 82.9%, and the NSCs still sustain the multi-differentiation potentiality [148].

Overall, efficient cryopreservation of neural tissue would be beneficial not only to potential clinical use but also for basic science and pharmaco-toxicology studies.

### Tissue-engineered constructs banking

Cryopreservation of tissue-engineered products by maintaining their structure and function is a prerequisite for large-scale clinical applications. Since the engineered tissue substitutes are undergoing clinical trials, and foreseeing a growing demand for cultured cells and tissues, the tissue engineering community is becoming increasingly worried about the challenge of providing sufficient amounts of these products to the market [161]. Cryopreservation is the only established mechanism for long-term preservation of cells, tissues and organs, as well as engineered tissues; therefore, it is a key step for the improvement of tissue engineering.

#### Cryopreservation of retinal organoids

Nakano et al. showed that an optimised vitrification method enables en bloc cryopreservation of stratified neural retina (NR) of human origin [162]. They developed an efficient cryopreservation method for storing stratified NR epithelia derived from human ESCs and found that a vitrification method with pre-treatment in 10% DMSO + 5% EG + 10% sucrose on ice was quite effective for NR cryopreservation. More recent findings show that both human iPSC-derived retinal organoids and dissociated retinal cells can be easily cryopreserved whilst retaining their phenotypic characteristics and the preservation of photoreceptor precursors [163].

#### Cryopreservation of bioartificial liver

Hepatocyte microbeads can temporarily replace the function of damaged hepatocytes in acute liver failure. Hence, intraperitoneal transplantation of hepatocyte microbeads is an attractive option for the management of acute liver failure. Interestingly, alginate-encapsulated hepatocyte spheroids were successfully cryopreserved by Massie et al. [164]. More recently, Jitraruch et al. developed an optimised cryopreservation protocol that could improve the outcome of cryopreserved hepatocyte microbeads for future clinical use [165].

#### Cryopreservation of decellularised oesophagi for tissue engineering

Efficient storage could potentially allow a timely use of decellularised oesophagi, essential for oesophageal tissue engineering and clinical translation. Recently, Urbani et al. published a protocol for long-term storage of decellularised oesophageal scaffolds for tissue engineering purposes which includes slow cooling with cryoprotectant solution in LN_2_ vapour [166].

#### Cryopreservation of tissue-engineered skeletal muscle

The tissue-engineered skeletal muscle plays an important role in the field of regenerative medicine and in emerging areas such as soft robotics, organ-on-a-chip disease models and drug testing [167]. Grant et al. published an optimised protocol in which the tissue-engineered skeletal muscle was frozen; tissue made from either differentiated myotubes or their undifferentiated myoblast precursors were investigated. After thawing the tissue maintained cell viability, and moreover, an optimised protocol in which the skeletal muscle was frozen undifferentiated, showed a threefold increase in force production as compared to unfrozen muscle [167].

#### Cryopreservation of engineered neural tissue

The ability to preserve engineered neural tissue without disrupting cellular and extracellular components and structures is important for clinical translation and commercialization. Of note, Day et al. studied the effect of cryogenic preservation on engineered neural tissue [168].

#### Cryopreservation of tissue-engineered pancreatic substitute

The use of encapsulated insulin-secreting cells represents a promising approach towards the treatment of insulin-dependent diabetes. Mukherjee et al. investigated cryopreservation of a model tissue-engineered pancreatic substitute by two ice-free cryopreservation (vitrification) solutions in comparison with a conventional freezing protocol and found vitrification to be a promising preservation procedure for this encapsulated cell system [169].

#### Cryopreservation of tissue-engineered bone

Yin et al. examined the feasibility of cryopreservation by vitrification of tissue-engineered bone composed of osteo-induced canine bone marrow mesenchymal stem cells and partially demineralized bone matrix scaffold and showed maintenance of cellular viability and osteogenic function after cryopreservation [170]. Recently, Tam et al. compared the effects of two potential preservation methods on the survival, quality and function of human tissue-engineered bone grafts from iPSCs. They found that storage at − 80 °C resulted in cell death and structural alteration of the extracellular matrix, whilst hypothermic storage at 4 °C did not significantly affect tissue viability and integrity [171].

#### Cryopreservation of tissue-engineered skin

Tissue-engineered epidermal membranes are useful for clinical wound healing. Chen et al. showed that when transplanted into nude mice, trehalose-cryopreserved artificial skin repaired skin defects in a similar manner to a non-cryopreserved control [172]. Moreover, they showed that trehalose-cryopreserved artificial skin resulted in engraftment and wound closure that was significantly enhanced compared with that of DMSO-cryopreserved epidermal membranes indicating that the use of trehalose improves cryopreservation of tissue-engineered epithelial sheets [172].

Cryopreservation of tissue-engineered human dermal replacement also plays an important role in skin tissue engineering and skin banking. An optimal cryopreservation protocol consisting of a cooling rate at 1 °C/min in 10% DMSO with the viability of studied dermal equivalent treated by this protocol being 75% of that of fresh control was derived by Wang et al. [173].

### Fertility cryopreservation

Fertility preservation (FP) is an important and rapidly growing field of reproductive medicine. Since 1978, when the first birth following in vitro fertilisation (IVF) was reported, at least 8 million babies have been produced with the help of medically assisted reproduction [174]. Cryopreservation techniques play a vital role in this treatment revolution, allowing the long-term storage of gametes and embryos without degradation of their quality for later use [174]. With a growing number of women postponing childbearing into their mid-thirties and beyond, the need for FP treatment has never been greater.

Beside elective FP available for healthy individuals, numerous medical conditions that compromise fertility require special attention in terms of FP. Particularly important is the need for FP in cancer patients. Most cancer treatments like chemotherapy, radiotherapy or a combination of both are highly toxic to the gonads and can cause infertility in both male and female patients. Non-malignant pathologies such as autoimmune diseases and their treatment, benign haematological diseases (most prominently β-thalassemia), gender dysphoria prior to starting hormone therapy, gynaecological or urologic and some genetic disorders (Turner’s syndrome, Fragile X syndrome, Klinefelter syndrome) could also lead to impaired fertility [175–179].

Continuous improvements in cancer treatment and early diagnosis significantly increased long-term survival rates in cancer patients. Cancer survival statistics are particularly impressive in children, with 80% of prepubertal cancer patients (0–14 years) likely to survive the disease [180, 181]. Survival rates in adolescent and young adult cancer patients (15–39 years) also improved dramatically in the last few decades [182]. Such an improvement in survival rates of cancer patients together with advancements in assisted reproductive technologies greatly increased the demand for FP for the childhood, adolescent and young adult patients [183].

#### Methods for male fertility preservation

Successful semen cryopreservation was achieved first, in the 1950s, thanks to their relative abundance for experimentation and small size [174]. Although slow cooling protocols can induce significant genetic damage, leading to low post-thaw mobility rate [184], freezing techniques for semen cryopreservation remain the most commonly used worldwide [174]. Comparative studies between slow cooling and vitrification showed that the use of vitrification techniques is less damaging for DNA, slightly improves post-warm motility rates and is more cost and time-efficient [185–187]. Several studies confirmed that long-term storage of sperm specimens (up to 40 years) did not affect the post-warm fertilisation of sperm [188–190]. However, a number of questions regarding the impact of specific cryopreservation protocols and suitability of donors are unresolved, and the research topic remains very active and clinically relevant [191].

For prepubertal boys who have not undergone spermatogenesis yet, and therefore not producing mature sperm, testicular tissue biopsy and spermatogonial stem cell cryopreservation is the only available option for fertility preservation for boys who are undergoing fertility reducing treatment such as chemotherapy. Germ cells are removed and frozen before treatment and are transplanted back into testes after treatment to restore spermatogenesis [192].

#### Methods for female fertility preservation

Embryo and oocyte cryopreservation are both well-established FP techniques. Embryo cryopreservation is the process of freezing and storing embryos at either cleavage or blastocyst stage. This technique has been proven as a safe and effective technology of FP over the last four decades. Since the first birth after embryo cryopreservation reported in 1983, more than half a million live births were achieved using this technique. Embryo cryopreservation represents the gold standard for FP due to high pregnancy rates [193, 194]. Importantly, human embryos preserved by vitrification showed higher survival rates post-warm than embryos preserved using slow cooling [195, 196].

Oocyte cryopreservation is the most widely used FP technique in the world for delaying childbirth [197]. Oocyte cryopreservation is technically more challenging than embryo cryopreservation due to oocyte high water content that makes it more prone to cryoinjury. Therefore, oocyte cryopreservation involves a higher risk of damage during the preservation process and results in lower overall pregnancy rates compared to embryo cryopreservation [198]. Some of the adverse effects of oocyte cryopreservation as summarised by Angarita et al. [193] are hardening of the zona pellucida [199], egg’s meiotic spindle, cytoskeleton and cortical granular damage by ice crystals [200]. Lower survival rates were reported after freezing of oocytes compared to vitrification as well as lower fertilisation rates [201]. When compared to natural conception, the incidence of congenital anomalies in born children does not differ significantly after cryopreservation [202].

Ovarian tissue cryopreservation is not a conventional FP technique but is the only available option for prepubertal girls and women who cannot delay the start of cancer therapy [203]. It involves harvesting and freezing of ovarian tissue allowing cryopreservation of oocytes within the primordial follicles presented in the ovarian cortex [193]. After thawing, ovarian tissue can be either transplanted back into a cancer survivor, or immature oocytes can be harvested and matured in vitro [194].

The first live birth after autotransplantation of previously frozen human ovarian tissue was reported in 2004 [204] with the number of live births exceeded 130 as of June 2017 [203]. The majority of these pregnancies occurred after natural conception, which is the main advantage of the ovarian tissue cryopreservation compared to oocyte and embryo cryopreservation [205]. Besides the restoration of fertility, the endocrine function of ovarian tissue was also successfully restored with the main duration of about 4–5 years after transplantation, with graft functioning up to several years [206], making this procedure an option to delay menopause [207].

### Stem cell cryopreservation

The commercial and clinical application of stem cells relies on cryopreservation as the only available long-term storage option. Stem cells represent highly promising resources for application in cell therapy and regenerative medicine, drug discovery, toxicology and developmental biology research [208]. Stem cell banking offers the opportunity to cryogenically preserve stem cells at their most potent state for later use [209]. Many stem cells come from once-in-a-lifetime tissues, like umbilical cord blood (see 5.5.1.); therefore, all over the world, stem cell banks are established in order to preserve these cells for their potential clinical application or future use in basic or translational research [208].

#### Umbilical cord blood (UCB) cryopreservation

Whilst UCB was treated as a medical waste in the past, nowadays, it is used to treat various diseases such as blood cancers as a valuable haematopoietic stem cell source. Since the first successful transplantation of cryopreserved UCB to treat an infant with Fanconi anaemia was performed in 1988 [210], the field of UCB banking and transplantation has grown exponentially and a global network of public and private biobanks of UCB was established [211]. By 2013, over 600,000 UCB units have been stored for transplantation worldwide, and more than 30,000 UCB transplantations have been performed [212].

#### Human pluripotent stem cells cryopreservation

Due to their unlimited propagation and differentiation abilities, human pluripotent stem cells including ESCs and iPSCs are also attractive types of stem cells for banking. Furthermore, iPSCs has opened up the potential for personalised cell therapies, and over the past decade, several initiatives have been established to collect and generate a large amount of human iPSCs for scientific research [213]. Many iPSC banks were established worldwide including the European Bank for induced pluripotent Stem Cells (EBiSC).

Other available stem cell sources for banking include bone marrow, umbilical cord tissue and adipose tissue. Dental stem cells are also an easily accessible source of multipotent adult stem cells [214] which makes them a suitable alternative for bone marrow-derived mesenchymal stem cells [215]. For that reason, dental tissues have become an attractive source of mesenchymal stem cells and dental stem cell banks are formed worldwide.

#### Haematopoietic stem cells cryopreservation

Haematopoietic progenitor cells (HPCs) are a highly relevant from a clinical point of view. Notably, allogeneic haematopoietic stem cell transplantation (HSCT) remains the only curative treatment for many blood disorders and is the most established stem cell treatment to date [216] Indeed, over the past 50 years, HSCTs have been performed in over 400,000 individuals. Along those lines, banking haematopoietic progenitor cells (HPCs) from the bone marrow, peripheral blood and umbilical cord blood is highly important for facilitating the finding of matched donors for patients needing HPC transplants. Cryopreservation of HPCs is the standard mode of handling for HSCT but has certain disadvantages as compared to the usage of non-cryopreserved HPCs [217]. Hence, further research is needed to improve the cryopreservation of this type of stem cells.

## Avenues for future research and applications

### Emergency medicine

Besides the medical applications described above that are already being explored, improved capabilities for artificially lowering the temperature of biological tissue could have a significant impact in many other fields. For instance, the possibility to gain more time is crucial in emergency medicine. Indeed, moderate states of hypothermia are common in clinical practice. Along those lines, therapeutic hypothermia has been associated with beneficial outcomes in patients following out-of-hospital cardiac arrest [218]. Moreover, animal studies demonstrate improved neurological outcome [219, 220]. Such capabilities would be also advantageous in warzones, where availabilities of equipment and medical staff are restricted, and so the extension of transfer time would have a big impact. Furthermore, cryopreservation of red blood cells is commonly used in settings where their availability is limited or unpredictable [221]. Despite these existent use cases, further research is required, e.g. to establish critical parameters in hypothermic emergency treatment, such as the duration, temperature and the substances to be administered.

### Space travel

Improved technologies for lowering the body temperature would also be of major benefit for space travel [222]. Currently, some of the most limiting factors for manned, long-duration space travel (e.g. to Mars) have to do with the requirements for keeping space travellers alive: beyond the availability of resources needed for normal physiology and metabolism, there are also significant health challenges posed by the long-term exposure to interplanetary radiation and zero-gravity, as well as the psychological stresses from confined space and associated social dynamics. Low-temperature biostasis or cryopreservation could render these issues negligible. Along those lines, the European Space Agency (ESA) and the US National Aeronautics and Space Administration (NASA) have both investigated the feasibility of suspended animation for deep-space travel [223–226]. Notably, it has been shown that rats (which are non-hibernators) can be synthetically induced into a hibernation-type state, namely torpor [227]. Torpor is a state of reduced metabolic rate and decreased body temperature. Intriguingly, it can protect from ionising radiation, further highlighting the potential benefits of this line of research for space travel.

### Quantitative methods

With the advances in computing power in the last decades, mathematical and computational approaches for analysing and modelling processes have gained substantial attraction in many biological fields (e.g. for omics, brain dynamics or synthetic biology research). Also in cryopreservation, quantitative methods have been useful to formalise complex physical dynamics. Along those lines, the seminal work of Mazur established that there is an optimal cooling rate for different cell types, which gave rise to mathematical equations describing the relationship between cell type-specific parameters and the optimal cooling rate [6, 228] or the addition and removal of CPAs [229]. More recently, such quantitative models have been used to inform on the timing of addition and removal of CPAs [230], as well as cooling rates and plunge temperature [231].

Importantly, computer models can be applied at various spatial scales and to varying detail: for instance, Weng et al. [232] made use of molecular dynamics simulations to assess the cryoprotective effect of different CPA concentrations and temperatures. Such modelling can help understand the impact of CPAs on a molecular level, and so potentially guide rational CPA design. Also making use of computational toolsets, but following a complementary approach, Solanki et al. [59] employ a computational model to study the volumetric properties of thermo-mechanical stress on a much larger spatial scale. Overall, it is anticipated that in the future, such computational methods will play an important role in the design of cryogenic processing protocols for medical applications, similar to current methods using computer-aided design tools, and so constitute a valuable tool in cryopreservation, which currently is mainly an experimental discipline.

## Concluding remarks

Cryopreservation is an interdisciplinary endeavour between medicine, biology, bioinformatics, chemistry and physics. The main challenges still to overcome are scaling up current methods to larger volumes and complex tissues. The larger the organ, or tissue volumes to be vitrified, correspondingly more time is required to cool and warm the organ. Not only thermal conductivity is an issue here but CPA viscosity limits for perfusion systems play a role. The protocols for cell lines, or even small tissues, such as sperm, eggs or corneas, cannot be replicated in larger human organs, which necessitate toxically high CPA concentration to inhibit ice formation during the longer time spent between the melting temperature and the glass transition temperature, and of course gives more time for toxic insults to accumulate [233]. The best techniques to get around these problems in small tissues use combinations of CPAs to reduce toxic effects of any single agent, using CPAs with weak water interactions to minimise disruption of hydration layers around biomolecules, using CPAs with mutual toxicity neutralisation effects, and reducing penetrating CPA concentrations by adding non-penetrating CPAs and ice blockers. Nonetheless, little is known about the mechanisms at work.

The challenges in cryobiology are not insurmountable. Future research will focus on ever more complex ways to prevent ice formation and mitigate cryoprotectant toxicity; novel cryoprotectants which exert disproportionately large cryoprotective effects compared to their concentration, in silico molecular modelling, and enhanced understanding of the processes that occur during cryopreservation will all be employed. One could envision a universal cryoprotectant solution, suitable for use in a range of tissue types, and physical advancements enabling high cooling and warming rates, or the manipulation of ice formation for large volume vitrification or freezing.

Whilst the concepts have been long known, the dedicated field of cryobiology dates back only around 70 years. In that time, it has advanced from freezing spermatozoa using glycerol, to vitrifying tissues, and even small organs using complex multi-component solutions. This is remarkable progress given that cryopreservation is as yet a relatively niche field of study, without garnering much attention in schools or undergraduate courses and utilising a fraction of the funding which is allocated to other causes. As such, there are still many opportunities that lie ahead, from short-term improvements in transplantation biology, to ambitions that may once have been viewed as science fiction, such as the building of organ banks or long-term suspended animation.

## Data Availability

Not applicable
